# A Japanese Encephalitis Virus Vaccine Inducing Antibodies Strongly Enhancing In Vitro Infection Is Protective in Pigs

**DOI:** 10.3390/v9050124

**Published:** 2017-05-22

**Authors:** Obdulio García-Nicolás, Meret E. Ricklin, Matthias Liniger, Nathalie J. Vielle, Sylvie Python, Philippe Souque, Pierre Charneau, Artur Summerfield

**Affiliations:** 1 Institute of Virology and Immunology, Sensemattstrasse 293, 3147 Mittelhäusern, Switzerland; obdulio.garcia-nicolas@ivi.admin.ch (O.G.-N.); meret.ricklin@gmail.com (M.E.R.); matthias.liniger@ivi.admin.ch (M.L.); nathalie.vielle@ivi.admin.ch (N.J.V.); sylvie.python@ivi.admin.ch (S.P.); 2 Department of Infectious Diseases and Immunopathology, Vetsuisse Faculty, University of Bern, Länggassstrasse 122, 3001 Bern, Switzerland; 3 Virologie Moléculaire et Vaccinologie, Institut Pasteur, 75015 Paris, France; pierre.charneau@pasteur.fr (P.S.); pierre.charneau@pasteur.fr (P.C.)

**Keywords:** Japanese encephalitis virus, antibody-dependent enhancement of infection, Fc receptor, lentiviral vector vaccine, vaccine-induced protection, persistence, mucosal virus shedding

## Abstract

The Japanese encephalitis virus (JEV) is responsible for zoonotic severe viral encephalitis transmitted by Culex mosquitoes. Although birds are reservoirs, pigs play a role as amplifying hosts, and are affected in particular through reproductive failure. Here, we show that a lentiviral JEV vector, expressing JEV prM and E proteins (TRIP/JEV.prME), but not JEV infection induces strong antibody-dependent enhancement (ADE) activities for infection of macrophages. Such antibodies strongly promoted infection via Fc receptors. ADE was found at both neutralizing and non-neutralizing serum dilutions. Nevertheless, in vivo JEV challenge of pigs demonstrated comparable protection induced by the TRIP/JEV.prME vaccine or heterologous JEV infection. Thus, either ADE antibodies cause no harm in the presence of neutralizing antibodies or may even have protective effects in vivo in pigs. Additionally, we found that both pre-infected and vaccinated pigs were not fully protected as low levels of viral RNA were found in lymphoid and nervous system tissue in some animals. Strikingly, the virus from the pre-infection persisted in the tonsils throughout the experiment. Finally, despite the vaccination challenge, viral RNA was detected in the oronasal swabs in all vaccinated pigs. These latter data are relevant when JEV vaccination is employed in pigs.

## 1. Introduction

Japanese encephalitis (JE), a mosquito-borne zoonotic viral disease endemic in parts of East Asia, Southeast Asia and Australasia, is considered the most important human viral encephalitis associated with fatality and severe sequelae [1,2,3,4]. Every year, 50,000 to 175,000 clinical JE cases in humans are reported, but it is estimated that less than 1% of infected people develop encephalitis [3,5]. Nevertheless, lethality in these cases can be up to 30%, and approximately 50% of surviving patients present long-term neurologic sequelae [3,5]. In addition, for pigs, JEV infection is of high relevance in endemic regions. Although the infection in adult swine is asymptomatic, it represents a significant cause of reproductive problems. Infection of pregnant sows can result in abortion, still-birth and birth defects. Furthermore, infected piglets can display fatal neurological disease [6].

The JE virus (JEV) is a positive single-stranded RNA virus belonging to the genus flavivirus, and encodes a polyprotein processed into three structural proteins being the capsid (C), the precursor membrane (prM), the envelope (E), and seven non-structural proteins (NS1-NS5) [4]. After virus assembly, virions undergo a maturation, in which prM is cleaved to generate M, and this process is required for viral entry into cells [4]. JEV is classified in five different genotypes G1–G5 [7,8]. In the last century, G3 was the dominant genotype and is now being replaced by G1 [9,10]. Recently, G5 strains have also re-emerged in China and South Korea [11,12].

Neutralizing antibodies targeting the E protein play a central role in immunological protection against JEV [13,14,15,16]. On the other side, antibodies have also been suspected to enhance disease in certain flavivirus infection, in particular, Dengue virus infection leading to severe hemorrhagic fever [17,18]. The proposed mechanism of antibody-dependent enhancement (ADE) of infection is based on virion-antibody complexes binding to Fc𝛾R expressing cells such as macrophages, resulting in enhancement of infection rather than neutralization [19]. In vivo ADE has also been described for flaviviruses closely related to JEV such as Murray Valley encephalitis virus in a mouse model [20]. There is also evidence that antibodies can enhance JE under certain conditions in a murine model [21].

Mosquitoes belonging to the Culex genus act as main vectors for JEV, while wild water birds represent the main vertebrate reservoir. Nevertheless, as pigs are highly susceptible to JEV infection and develop a relatively high viremia for several days, they can play an important role in the ecology of the virus as amplifying hosts [1,3,8,22]. This contrasts with horses and humans which may develop fatal disease but do not contribute to further transmission of JEV to mosquitoes or other species [3]. Considering this situation, vaccination of pigs against JEV is and has been widely practiced in certain countries such as Japan and South Korea [6]. 

Up to now, JEV remained endemic mainly in Southeast Asia but climate warming, globalization and virus adaptation to new arthropod vectors could result in the emergence of JEV in other parts of the world, as it has occurred for West Nile virus and Zika virus. Furthermore, we have recently shown that, under experimental conditions, the virus can transmit between pigs by contact and is secreted in oronasal fluids for a prolonged period of time [23]. This indicates a potential of JEV to spread and circulate even in areas with a climate unfavorable to the virus transmission by mosquitos. An additional concern is that JEV has a particularly high tropism for the tonsils and can persist in this tissue for several weeks [23,24], providing a possible mechanism of virus overwintering in the pig population. For these reasons and because vaccination represents an efficient countermeasure against JEV, we have recently developed a novel vaccine based on a lentiviral TRIP/JEV which expressed JEV G3 prM and E proteins (TRIP/JEV.prME) [25]. 

Considering the possible involvement of ADE during flavivirus infections, the present study investigated ADE activities of sera from TRIP/JEV.prME-immunized compared to JEV-infected pigs. To our surprise, we found particularly high levels of ADE with sera following TRIP/JEV.prME vaccination. This in vitro ADE of infection was found with macrophages and an Fc receptor (FcR) expressing kidney cell line. However, despite these responses, the TRIP/JEV.prME vaccine was found to induce protective immunity as demonstrated in a heterologous challenge infection in pigs. 

## 2. Materials and Methods

### 2.1. Monocyte-Derived Macrophages

Blood was obtained from specific pathogen free (SPF) Swiss Large White pigs. The blood sampling was approved by the cantonal ethical committee for animal experiments, license #BE88/14. Peripheral blood mononuclear cells (PBMC) were isolated using ficoll-paque density centrifugation (1.077 g/L; GE Healthcare Life Sciences, Dübendorf, Switzerland). Monocytes were sorted as CD172a+ cells using monoclonal antibody (mAb), clone 74-22-15A (hybridoma kindly provided by Dr. A. Saalmüller, Veterinary University of Vienna, Austria) and magnetic cell sorting with LS columns and the MACS (magnetic cell sorting) system (Miltenyi Biotec GmbH, Bergisch Gladbach, Germany). Macrophages were generated as previously described [26]. Briefly, monocytes were cultured at 5 × 10^5^ cell/mL in Dulbecco’s modified Eagle’s medium (DMEM) containing Glutamax (ThermoFisher Scientific, Zug, Switzerland) supplemented with 10% of specific pathogen-free porcine serum (produced in-house), seeded in 24-well culture plates and incubated for three days at 39 °C and 5% CO_2_.

### 2.2. Generation of CD16 Expressing SK6 Cells

For generation of CD16 expressing SK6 cells, a lentivirus (LV) expression system using plasmids obtained from the laboratory of Dr. Didier Trono (Ecole Polytechnique Federale de Lausanne, Switzerland) or through Addgene (Cambridge MA, USA) [27,28] was employed to co-express FcγRIIIa and the common γ-chain. This is required for stable expression of CD16 on the cell surface (unpublished results). Porcine FcγRIIIa (FCGR3A, GenBank AF372453.1) and porcine FcR common γ-chain (FCER1G; NCBI NM_001001265.1) were cloned into the lentiviral transfer plasmid pWPT-GFP. FCGR3A and FCER1G were amplified from cDNA obtained from porcine PBMCs using the oligonucleotides pCD16_F (5′-CTACCTACGCGTCACCATGTGGCAGCTGCTGTCACC-3′) and pCD16_R (5′-TGCCGTCGACTTATCCTCCTTTGTCCTGCGG-3′) or pFceRI_F (5′-CTACCTACGCGTCACCATGATTCCAGCAGTGGTCTTGC-3′) and pFceRI_R (5′-TGCCCTCGAGTTACTGTGGTGGTTTCTCATGC-3′). The MluI and SalI fragment containing FCGR3A and the MluI and XhoI fragment containing FCER1G were cloned into the MluI and SalI sites of the pWPT-GFP vector, resulting in pWPT-FCGR3A and pWPT-FCER1G. The nucleotide sequences of the plasmid inserts were verified by automated DNA sequencing using the ABI 3130 Genetic Analyzer (Life Technologies, Zug, Switzerland).

In order to generate two different lentiviruses (expressing FCGR3A or FCER1G), HEK293T cells were transfected with the envelope plasmid (pMD2.G), the packaging plasmid (pCMV-R8.74) and the pWPT-FCGR3A or pWPT-FCER1G plasmids using standard calcium phosphate precipitation. Medium was changed after overnight incubation at 37 °C and the supernatant harvested after 48 h, centrifuged (350× *g*, 10 min) and filtered. The virus was purified and enriched by centrifugation on a 20% sucrose cushion at 100,000× *g* for 90 min at 4 °C. SK6 cells were transduced twice with 1:100 dilutions of the purified lentiviruses in 1 mL serum free medium of a T25 cell culture flask followed by culture overnight at 37 °C and medium change between the transductions. After 5 days, cells were stained with anti-CD16 mAb G7 (Becton Dickinson, Basel, Switzerland) and sorted by flow cytometry (FACSAria, Becton Dickinson) to obtain >95% pure CD16+ SK6 cells. The cells termed SK6-CD16 were then expanded and stored in liquid nitrogen for further proliferation. CD16 expression was found to remain stable over at least five passages.

### 2.3. Viruses

The following JEV strains were used: JEV Laos (G1; CNS769_Laos_2009; [23,29]) kindly provided by Prof. Remi Charrel, Aix-Marseille Université, Marseille, France; JEV Nakayama strain (G3; National Collection of Pathogenic Viruses, Salisbury, UK); JEV S-g5/NS-g3, which represents a chimeric G3/G5 expressing the structural proteins of the G5 strain XZ0934 fused to the nonstructural proteins of JEV G3 RP-9 [25], was kindly obtained from Dr. Philipp Despres, Université de La Réunion, France). All JEV strains were propagated in Vero cells in G-MEM BHK-21 medium (ThermoFisher Scientific) supplemented with 2% fetal bovine serum (FBS; Biowest, Nuaillé, France) and cultured at 37 °C and 5% CO_2_. Virus titrations were determined using Vero cells. Infected cells were detected using immunoperoxidase monolayer assay (IPMA) with the anti-flavivirus E mAb 4G2 (ATCC). Titers were calculated and expressed as 50% tissue culture infective dose per mL (TCID50/mL). 

### 2.4. Antibody-Dependent Enhancement of Infection

A collection of sera from previously published work was employed (Table 1). This included sera from pigs vaccinated with the lentiviral vector-based vaccine expressing prM and E of G5 strain XZ0934 (TRIP/JEV.prME) [25]. In addition, we also used sera from pigs infected with JEV G1 Laos and G3 Nakayama strains and collected at 11 days post infection (p.i.) [24]. As negative control, naïve serum from SPF pigs was included.

To test the ADE of these sera, different serum dilutions were incubated during 30 min at 37 °C with an equal volume of viral suspension at a dose of 0.1 TCID_50_/cell, followed by addition to porcine macrophages or SK6-CD16 cells. To verify JEV strain-dependent differences, ADE of infection mediated by the anti-flavivirus E protein mAb 4G2 was tested using the murine J744A.1 macrophages cell line (ATCC, cultured in DMEM supplemented with 10% FBS). After incubation for 1 h at 37 °C, the cells were washed and fresh medium was added. After 24 h, the cells were then analyzed for expression of JEV E protein using flow cytometry. To this end, cells in suspension were fixed with 4% (*w*/*v*) paraformaldehyde during 10 min at room temperature, followed by washing and permeabilization with 0.3% (*w*/*v*) saponin in PBS in presence of anti-flavivirus E protein mAb 4G2 for 15 min on ice. After washing, anti-mouse Alexa 647 fluochrome conjugate (ThermoFisher Scientific) was added for 15 min and the cells were acquired on a FACSCantoII (Becton Dickinson). For analysis, Flowjo V.9.1 software (Treestars, Inc., Ashland, OR, USA) was used. Dead cells were excluded by electronic gating in forward/side scatter plots, followed by exclusion of doublets. 

### 2.5. Vaccination Challenge Experiment

The pig immunization/challenge experiment was conducted according to Swiss animal welfare regulations and approved by the cantonal ethical committee of Bern (approval number BE 118-13). Five-week-old SPF Swiss Landrace piglets from our own in-house breeding were randomly allocated into three different groups of three animals each. Prior to the first immunization, the animals were left one week for adaptation. The first group was immunized with the TRIP/JEV.prME lentiviral vector produced as previously described [25]. These pigs received 10^5^ transduction units (TU) diluted in 0.5 mL in DMEM intramuscularly, followed by booster immunization after three weeks. The second group was intradermally inoculated with JEV G1 Laos at 10^5^ TCID_50_ diluted in DMEM. The third group of animals was intradermally inoculated with DMEM as control. The sera were collected before vaccination/infection, and then once a week. Thirty-six days after the first TRIP/JEV.prME vaccination or JEV Laos infection, all pigs were challenged with JEV G3 Nakayama at 10^3^ TCID_50_ using oro-nasal administration. Thereafter, clinical signs and body temperature were checked and blood taken daily until the end of the study at 10 days post-challenge.

### 2.6. Virological Analyses

For reverse transcription quantitative polymerase chain reaction (RT-qPCR) based quantification of viral RNA, 1.5 mL tubes were filled with 500 μL of minimum essential medium (MEM; ThermoFisher Scientific) and weighed before and after filling of the organs samples. Samples were lysed with a BulletBlender (Next Advanced Inc., Averill Park, NY, USA), and after centrifugation, the supernatants were transferred into new tubes and immediately frozen at −80 °C for storage. After thawing, each sample was spiked with a defined amount of enhanced green fluorescent protein (eGFP) RNA. Then, RNA extraction was performed using the QIAmp viral RNA extraction kit (Qiagen AG, Hombrechtikon, Switzerland) following the manufacturer's instructions. RT-qPCR of the highly conserved 3′ NTR of the JEV genome was performed as previously described [30]. With the aim to discriminate between JEV G1 Laos and JEV G3 Nakayama strains, specific sets of primers and probes were designed and RT-qPCR conditions optimized (Table 2). RT-qPCR used the SuperScript III Platinium One-Step qRT-PCR system (ThermoFisher Scientific) with ROX (carboxy-x-rhodamine) reference dye according to manufacturer’s instructions, and where run in a 7500 Applied Biosystems Real-time PCR machine (ThermoFisher Scientific). The thermal cycling setup was 30 min at 50 °C for the RT step, then qPCR steps which included 2 min at 95 °C for enzyme activation, and 50 cycles of denaturation at 95 °C during 15 s, annealing at 60 °C for 30 s and extension at 72 °C for 30 s. Samples were taken as positive only with the cycle threshold (CT) value of the internal eGFP control was lower than 28. Viral load was quantified relatively by using RNA from a stock of Nakayama JEV with a known titer as a standard. The stock was serially diluted tenfold, RNA was extracted, and CT values were determined to draw a standard curve (correlation coefficient R = 0.99). CT values above 40 were defined as negative. The CT value corresponding to 1 TCID50 was defined as 1 RNA unit (U). Using this standard, the CT values of our samples were transformed into relative quantities as RNA U/mL. Organ samples were corrected for their weight and data calculated as relative RNA quantities in U/mg.

### 2.7. Serum Neutralization Assay

Neutralizing antibodies against JEV were determined by focus reduction neutralization test (FRNT) on Vero cells as previously described [25]. Briefly, pig sera were two-fold serially diluted starting in 1:5 serum dilution, and incubated with 100 focus-forming units (FFU) of JEV for 30 min at 37 °C and then added to Vero cells for 1h at 37 °C. After removal of the inoculum and washing once, DMEM supplemented with 2% FBS was added and culture at 37 °C. Infected cells were fixed, permeabilized and stained as described above using the 4G2 mAb. The highest serum dilution which reduced the FFU by 50% was defined as the end-point titer and expressed as FRNT_50_/mL.

### 2.8. Statistics

Statistical analyses for the ADE of infection were tested using two-way ANOVA followed by Dunnets’s multiple comparison test (variables were serum dilution and serum origin). For neutralizing antibodies, data was Log2 transformed and *p*-values determined using two-way ANOVA and Sidak’s multiple comparison (variables were time p.i. and serum origin). All tests were made with GraphPad Prism 7 (GraphPad Software version 7.0b, La Jolla, San Diego, CA, USA). Alpha was set to 0.05; * *p* < 0.05, ** *p* < 0.002, *** *p* < 0.001. 

## 3. Results

### 3.1. TRIP/JEV.prME Induces ADE of Macrophage Infection

**Figure 1:**
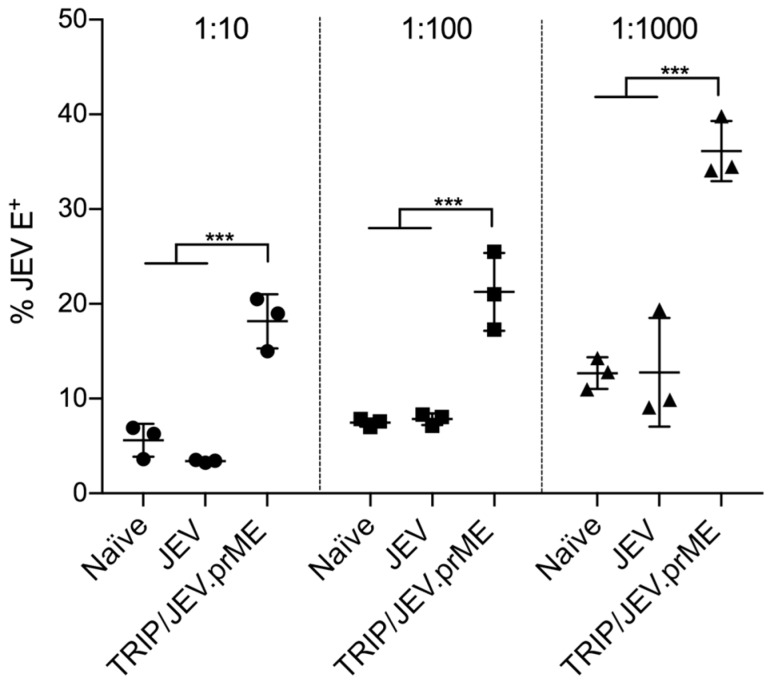
Antibody-dependent enhancement (ADE) of macrophage infection. Sera from piglets immunized with lentiviral vector TRIP/JEV which expressed JEV G3 prM and E proteins (TRIP/JEV.prME) or infected with Japanese encephalitis virus (JEV) G3 Nakayama (focus 50% reduction neutralization test; FRNT50 1:160 and 1:320, respectively) were 10-fold diluted (from 1:10 to 1:1000) and incubated with JEV G3 Nakayama at multiplicity of infection (MOI) 0.1 of 50% tissue culture infective dose per mL (TCID50) per cell during 30 min at 37 °C, and then added to the cells. The percentage of cells expressing JEV E protein as a measure of ADE of infection in macrophages is shown. Statistical significance was calculated using a two-way ANOVA followed by Dunnets’s multiple comparison. The results are representative of triplicate cultures repeated in three independent experiments. * *p* < 0.05, ** *p* < 0.002, *** *p* < 0.001.

To test a possible ADE of infection in macrophages, sera from TRIP/JEV.prME-immunized and JEV-infected pigs were incubated at different concentrations with JEV G3 Nakayama, and infectivity tested for monocyte-derived macrophages. Our results demonstrated that, while no statistically significant ADE was found with the immune serum from the JEV-infected animals, sera from TRIP/JEV.prME-immunized animals (FRNT_50_ 1:160) strongly promoted infection by JEV, even at high dilutions (Figure 1). 

### 3.2. TRIP/JEV.prME-Antibodies Strongly Enhance JEV Infection of Cells Expressing FcγRIII

**Figure 2:**
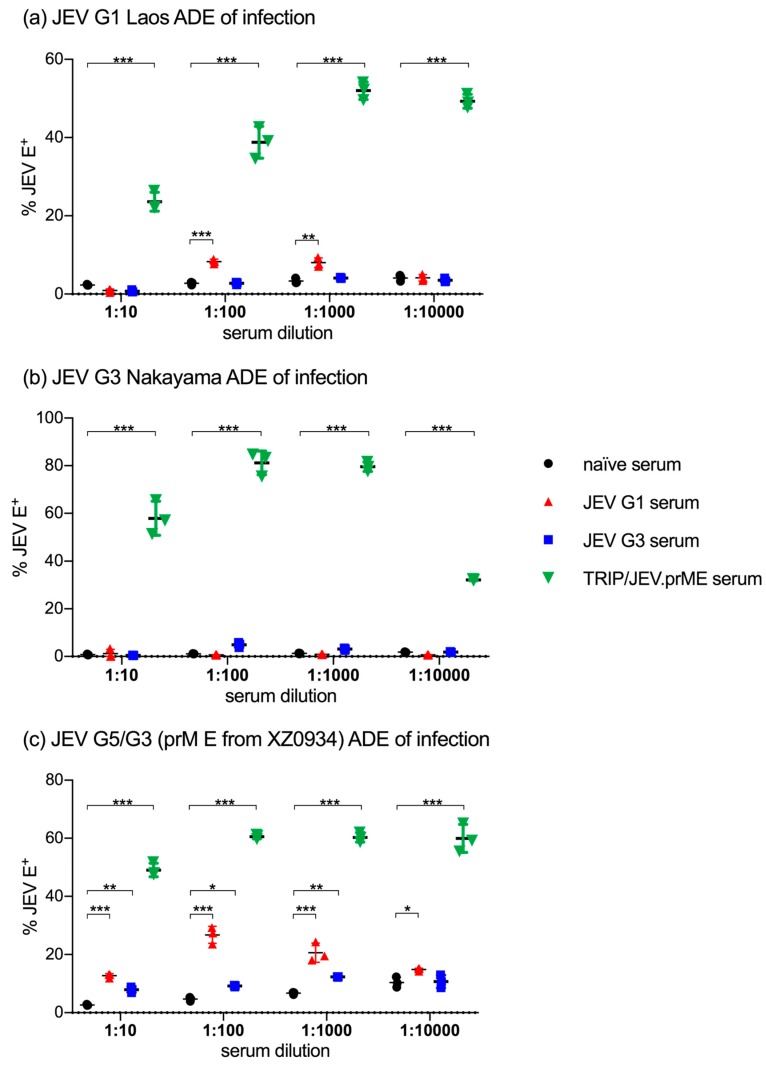
ADE of infection in SK6-CD16 cells. Sera from TRIP/JEV.prME-immunized, JEV Laos- and JEV Nakayama-infected pigs were tested for ADE activity in the porcine kidney cell line SK6 expressing CD16. ADE of infection was tested as described in Figure 1 using (**a**) JEV G1 Laos; (**b**) JEV G3 Nakayama and (**c**) JEV G5/G3, representing a chimeric virus expressing a G5 prM/E. The percentage of infected cells was determined after 24 h. Statistical significance was calculated using a two-way ANOVA followed by Dunnets’s multiple comparison. The results are representative of triplicate cultures repeated in two independent experiments. * *p* < 0.05, ** *p* < 0.002, *** *p* < 0.001.

Considering the ADE of infection by JEV opsonized with TRIP/JEV.prME serum in macrophages, we tested if similar observations could be made using the porcine kidney cell line SK6 engineered to express porcine FcγRIII (SK6-CD16). To this end, we compared sera from TRIP/JEV.prME-vaccinated pigs with sera from pigs infected with JEV G1 (Laos) and G3 (Nakayama). These sera were tested against JEV G1 (Figure 2a), JEV G3 (Figure 2b) and against JEV G5/G3 (homologous prM/E to the TRIP/JEV.prME vector).

The data obtained confirmed the very potent ADE activity of the TRIP/JEV.prME sera in enhancing infection with all three JEV genotypes. Its efficiency was also demonstrated by the fact that ADE was even seen at serum dilutions of 1:10,000 although the neutralizing titers of this serum was 1:40 against JEV G1 Laos and 1:160 against both JEV G3 Nakayama and the chimeric G3/G5 JEV [25]. Only for the Nakayama strain was there was a clear reduction of ADE at this serum dilution (Figure 2b).

In contrast to this, the ADE activity of various sera from JEV-infected pigs was absent or much lower. Anti-JEV G1 Laos serum had a moderately but statistically significant ADE activity for a homologous virus (Figure 2a), no activity of JEV G3 Nakayama (Figure 2b), but relatively strong ADE activity for the G5/G3 chimeric virus (Figure 2c). Anti-JEV G3 Nakayama serum had no ADE activity for JEV G1 Laos (Figure 2a) and the homologous virus (Figure 2b), but significantly enhanced infection by G5/G3 JEV (Figure 2c). These results were in accordance with experiments investigating the ability of the anti-flavivirus E protein mAb4G2 to enhance infection of murine J744A.1 macrophages. The strongest ADE activity was found with the JEV G5/G3 chimeric virus and no enhanced infection by JEV G3 Nakayama (Figure S1).

There results indicate that also viral factors, which are independent of the antigenic relationship to the serum, determine the infectivity of opsonized virus. On the other hand, the fact that Laos-immunized but not Nakayama-immunized pigs developed ADE antibodies against a homologous virus, demonstrates strain-dependent differences in the ability to induce ADE antibodies in pigs. 

### 3.3. Antibody Responses Induced by TRIP/JEV.prME Vaccine and JEV Infection

Considering the strong ADE of infection induced by the TRIP/JEV.prME vaccine but not following JEV infection, we decided to compare the protection induced by the vaccine to that following JEV G1 Laos infection. We selected G3 Nakayama strain as a challenge virus as ADE of infection by this virus was only enhanced with TRIP/JEV.prME antisera (Figure 2b). 

**Figure 3:**
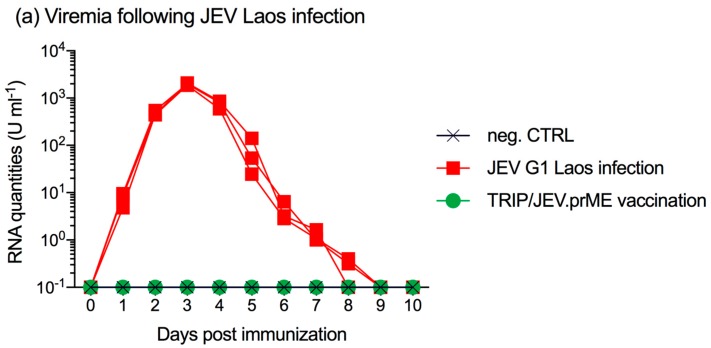

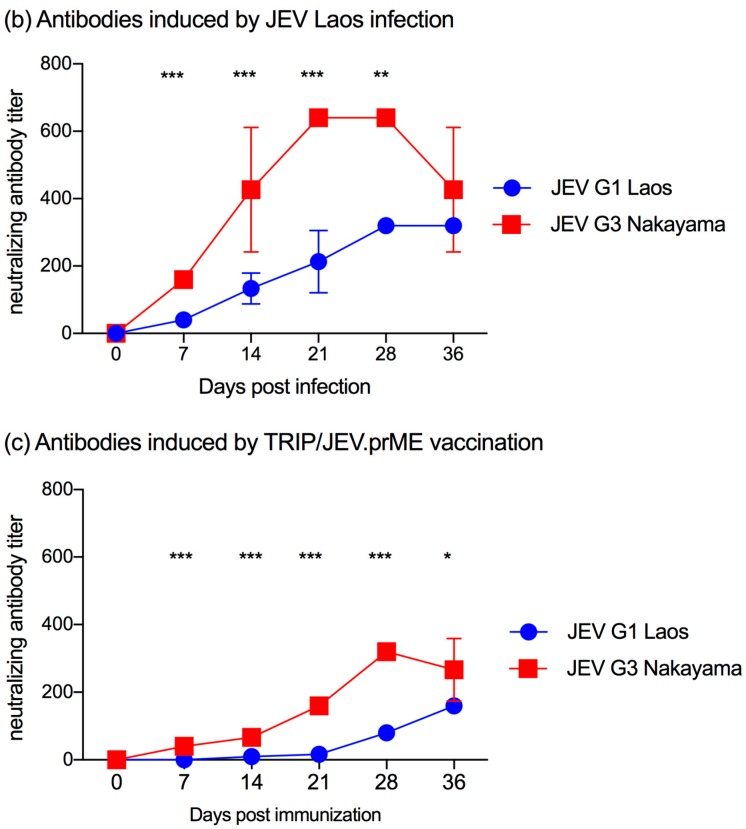
Neutralizing antibody response in piglets immunized with JEV G1 Laos or TRIP/JEV.prME. Groups of three pigs were either infected with JEV Laos or immunized with TRIP/JEV.prME or mock-inoculated, and serum was collected at the indicated time points (*x*-axis). (**a**) viral RNA load determined by RT-qPCR in sera from all nine animals; (**b**) JEV Laos- and (**c**) TRIP/JEV.prME-induced neutralizing antibody responses of sera against homologous JEV Laos (blue circles) and JEV Nakayama (red squares). Mean and standard deviations are shown. Statistical significance was determined after Log2 transformation of the data using two-way ANOVA and Sidak’s multiple comparison. * *p* < 0.05, ** *p* < 0.002, *** *p* < 0.001.

All three pigs infected with JEV G1 Laos strain became viremic as early as one day p.i. and remained positive for viral RNA until 7–8 days p.i. (Figure 3a), comparable to previously published results [23]. 

All three animals seroconverted after one week and developed serum neutralizing antibodies against homologous and heterologous JEV G1 Laos and G3 Nakayama strains (Figure 3b). This coincided with the end of the viremia. Between days 14 and 28 p.i., the neutralizing antibodies further increased. Surprisingly, at these time points, titers were even higher against the heterologous JEV G3 strain. Nevertheless, at the time of challenge infection (day 36), there was no statistical significance between the neutralization of the Laos and Nakayama strains. 

Piglets immunized with TRIP/JEV.prME lentiviral vector also developed neutralizing antibodies but at a slower and weaker rate (Figure 3c). Again, neutralization activity against the Nakayama strain was found to be more potent than against the Laos strain. At day 36 post vaccination, titers were between 160 and 320 with all pigs.

### 3.4. TRIP/JEV.prME Vaccine and Previous JEV Infection Induce Protection against Viremia

**Figure 4:**
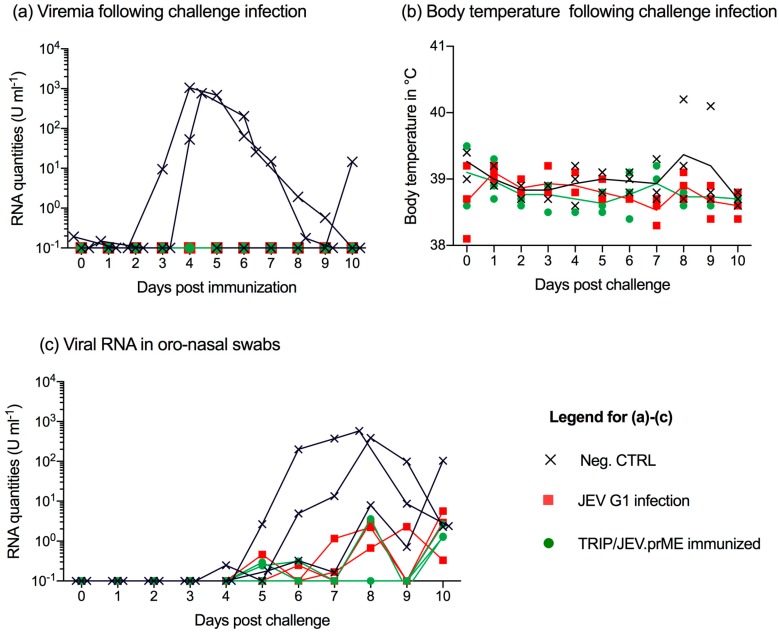
TRIP/JEV.prME vaccine and previous JEV infection induce protection but do not prevent virus shedding. Groups of three pigs were either mock-inoculated (Neg. CTRL, cross), immunized by previous infection with JEV Laos (red square) or vaccinated with TRIP/JEV.prME (green circle), and then challenged infected with JEV G3 Nakayama. Data post challenge is shown. (**a**) viral RNA loads determined by RT-qPCR in sera from all nine animals; (**b**) body temperature; (**c**) viral RNA load in oro-nasal swabs collected daily.

At day 36 post vaccination, all nine animals were challenge infected with JEV G3 Nakayama. Only animals from the unvaccinated control group developed viremia in terms of viral RNA detection in the serum. This started in two pigs at 3–4 days p.i. and lasted for 4–6 days. In one animal, viremia was only found 10 days p.i. (Figure 4a). The virus infection did not induce clinical signs with the exception of fever in one of the control animals at days 8 and 9 p.i. (Figure 4b).

### 3.5. JEV Immunization Does Not Completely Prevent Oro-Nasal Shedding of Challenge Virus

Considering the ability of JEV to shed through oro-nasal secretions, which can result in vector-free transmission by contact [23], we collected oro-nasal swabs and tested them by RT-qPCR (Figure 4c). Animals from the control group shed virus from 4 to 10 days p.i. Interestingly, we detected low but clearly detectable JEV RNA in many oro-nasal swabs samples from all pigs previously infected with JEV G1 Laos or vaccinated with TRIP/JEV.prME (Figure 4c).

### 3.6. JEV Immunization Does Not Provide Sterile Immunity

**Figure 5:**
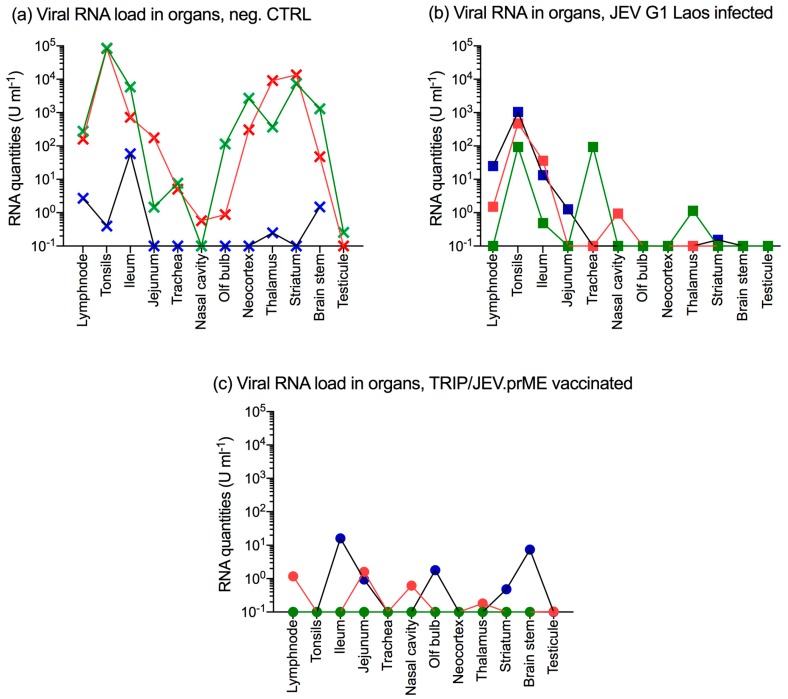
TRIP/JEV.prME vaccine or previous JEV infection induce protection but do not prevent organ infection. Groups of three pigs were either unvaccinated/infected (negative control; Neg. CTRL, crosses), immunized by previous infection with JEV Laos (squares) or vaccinated with TRIP/JEV.prME (circles), and then challenge infected with JEV G3 Nakayama. At 10 days p.i., the animals were euthanized and organ samples tested for viral RNA by RTqPCR. (**a**) viral RNA load in pigs from the Neg. CTRL group; (**b**) viral RNA load from the JEV Laos pre-infected group; (**c**) viral RNA load from the TRIP/JEVprME-vaccinated group.

At 10 days p.i., all animals were euthanized and various tissues analyzed for viral RNA. In the negative control group, the two viremic animals had high levels of JEV RNA in lymphoid tissues including the tonsils, the lymph node and the continuous Peyer’s patches of the terminal ileum (Figure 5a). Similar to previous studies [23,24], these pigs also had high viral RNA quantities in the neocortex, the thalamus and the striatum. The third animal in this group, which only became viremic at 10 days p.i., also had viral RNA in lymphatic tissues, thalamus and brain stem but reaching much lower levels (blue crosses).

In the JEV G1 pre-infected group, viral RNA was found in the tonsils and ileum of all pigs and in lymph nodes of two pigs. Furthermore, one animal also had a virus in the jejunum, the trachea, nasal cavity, although at very low levels (Figure 5b). In pigs immunized with TRIP/JEV.prME lentiviral vector, low levels of viral RNA were found in the lymph node, ileum, jejunum, nasal cavity, olfactory bulb, striatum and brain stem. One animal was negative in all tissues (Figure 5c).

### 3.7. JEV G1 Persistence after JEV G3 Challenge

Considering the relatively high viral loads in the tonsils found in the JEV Laos-preinfected group, and the previously described ability of JEV to persist for several weeks in the tonsils [23], we re-analyzed these samples with a set of primers and probes which discriminate between JEV G1 Laos (first infection) and JEV G3 Nakayama (challenge virus). For all three animals, these strain-specific RT-qPCR’s were only positive for JEV G1 Laos demonstrating the long-term persistence for least 46 days, even following challenge infection with a heterologous JEV strain. As expected, only Nakayama-specific viral transcripts were found in the unvaccinated (Neg. CTRL) group (Table 3).

Considering these results, we also re-tested all swabs using the strain-specific RT-qPCR. Interestingly, only the RT-qPCR detecting JEV G3 Nakayama was positive, demonstrating that the shed virus was originating from the challenge infection.

## 4. Discussion

In the present study, we have discovered that the TRIP/JEV.prME, in contrast to JEV infection, induced very high levels of antibodies with ADE activity. Considering the possible importance of ADE of disease during certain flavivirus infections, we decided to test the protective value of this vaccine and found it to be at least as protective as a previous JEV infection. 

For flaviviruses, ADE can occur through various mechanisms. Using West Nile virus (WNV) as model, it was demonstrated that ADE may occur when antibodies concentration does not achieve the minimum stoichiometric threshold for viral neutralization. This explains that ADE is often seen at sub-neutralizing antibody concentrations [31]. Nevertheless, the sera from the TRIP/JEV.prME immunized animals also strongly enhanced infection at concentrations clearly above the neutralization titers. An alternative explanation would be that the TRIP/JEV.prME vaccine induces antibodies with a particular specificity causing high ADE activity. For instance, antibodies against the prM protein are known to mediate ADE [32,33]. In fact, our previous work showed that TRIP/JEV.prME immunized animals developed antibodies against both E and prM proteins [25]. Alternatively or additionally, the TRIP/JEV.prME vaccine may induce antibodies against the fusion loop of the E protein, also known to cause ADE [34]. We also have no indication that the strong ADE activity could be related to differences in antibody isotypes induced by the TRIP/JEV.prME vaccine, as these were similar to those from JEV infected pigs [25]. Future studies would be required to identify the targets of the antibodies induced by the TRIP/JEV.prME vaccine. 

In our study, we also observed differences in the susceptibility of three different JEV virus strains to ADE. It is well known that immature virions express prM on their surface, making them susceptible to ADE [35]. Although the cleavage of prM is important for maturation of virions to full infectivity, this process is often incomplete, showing substantial variability between viruses [36]. Therefore, virus preparations are typically a mixture of immature and mature virions, and even the passage history of a virus can have an effect on in vitro ADE [31,37]. These possible differences in virus structure between strains also influence virus neutralization [38], which may explain why the heterologous virus was more efficiently neutralized in the present work. For this study, we used virus preparations produced in Vero cells, described to produce many immature virions expressing prM [39,40]. 

The observation that the TRIP/JEV.prME vaccine protects is in line with other studies showing that antibodies causing ADE in vitro can be protective. This has been demonstrated for antibodies against the fusion loop in a WNV murine model [41]. Furthermore, no association was found between the levels of anti-prM antibodies and the severity of Dengue in human beings [42].

The in vivo trial performed in this study confirmed and complemented two important findings related to JEV infection in pigs. First, we confirmed that JEV can persist long term in the tonsils of infected pigs as previously described [23]. In the present study, persistence was found for at least 46 days, even after a second heterologous challenge infection expected to boost antiviral immunity. This indicates that the virus is well hidden from neutralizing antibodies and cytotoxic T-cells. Second, we also confirmed the oro-nasal virus shedding peaking clearly after the viremia. This means that the highest degree of shedding occurs when the pigs are basically no longer viremic. We also found a low degree of virus shedding in vaccinated animals, although these were never viremic. It appears that the source of the virus detected in the oronasal swabs was not the tonsils. The virus detected in the tonsil was exclusively JEV Laos G1 utilized for the first immunization, whereas only the challenge virus (Nakayama) was found in the swabs. Clearly, more research addressing the source of the virus in oronasal secretion, which is probably local, is required. 

In accordance with our previous work, experimental JEV infection under our defined conditions induced no or only mild signs of disease [23,24]. In the present study, the animals were also older compared to our previous work explaining the complete lack of clinical signs. Nevertheless, similar to previous results, JEV RNA was readily detected in CNS tissues and lymphoid tissues with the highest viral RNA loads were found in the tonsils. An important observation was also that previous infection or vaccination did not prevent secondary infection of pigs. Even if the oro-nasal viral shedding was low in immunized pigs, these animals may still be able to transmit the virus to other pigs in close contact. This is based on our work showing that 10 TCID_50_ given oro-nasally is sufficient to infect pigs [23]. Future studies are required to address if transmission can occur under such conditions and the role of contact transmission in field situations.

## 5. Conclusions

The present study demonstrates that a viral vector vaccine based on prM and E protein expression induces high levels of antibodies that strongly enhance infection of FcγR expressing cells, but still provides protection comparable to a natural infection. This has implications for vaccine design against JEV and other flaviviruses. Furthermore, our data on virus persistence and shedding are of relevance for JEV ecology and pig vaccination.

## Figures and Tables

**Table 1:** Sera collection employed for antibody-dependent enhancement (ADE) experiments.

Serum	JEV Strain	FRNT_50_
JEV G1 antisera	G1	1:80
	G3	1:320
	G3/G5	1:160
JEV G3 antisera	G1	1:20
	G3	1:320–640
	G3/G5	1:80
TRIP/JEV.prME antisera	G1	1:40
	G3	1:160
	G3/G5	1:60

JEV: Japanese encephalitis virus; FRNT_50_: focus 50% reduction neutralization test.

**Table 2:** Primer and probe sets employed for JEV reverse transcription-quantitative PCR (RT-qPCR).

Specificity		Primer and Probe Sequence (5′-3′)	Concentration (nM)
3′ NTR JEV	forward	GGTGTAAGGACTAGAGGTTAGAGG	200
	reverse	ATTCCCAGGTGTCAATATGCTGTT	200
	probe	FAM-CCCGTGGAAACAACATCATGCGGC-BHQ-1	100
JEV G1 Laos	forward	GACAGGATAAAGTCATGTGCGT	200
	reverse	CCTGACGTTGGTCTTTCAAC	200
	probe	FAM-CCGTCTCGGAAGCAGGTCCC-BHQ-1	100
JEV G3 Nakayama	forward	CAGGGTCATCTAGTGTGATTTAAGG	1600
	reverse	CAGTCCTCCTGGGACTGAGA	1600
	probe	FAM-TGCTGGCCTGACTCCATATGCA-BHQ-1	200

Infectious virus quantification was determined by titration on Vero cells as previously described [23].

**Table 3:** JEV Nakayama and JEV Laos viral RNA loads in tonsils at 10 days post challenge

Group/Pig Number ^1^	Nakayama-Specific RT-qPCR	Laos-Specific RT-qPCR
Neg. CTRL, #1516	5.2 × 10^0^	negative
Neg. CTRL, #1517	4.6 × 10^5^	negative
Neg. CTRL, #1518	2.0 × 10^5^	negative
JEV G1, #1512	negative	3.2 × 10^1^
JEV G1, #1513	negative	1.7 × 10^1^
JEV G1, #1521	negative	2.9 × 10^0^

^1^ groups as defined in Figure 4.

## Supplementary Materials

The following are available online at www.mdpi.com/1999-4915/9/5/124/s1, Figure S1: ADE of infection in murine macrophages J744A.1 cells.
