# Supplementary material for: A Japanese Encephalitis Virus Vaccine Inducing Antibodies Strongly Enhancing In Vitro Infection Is Protective in Pigs
Source: Viruses. 2017 May 22;9(5):124. doi: 10.3390/v9050124 (PMC5454436; doi:10.3390/v9050124)
Supplement: Supplementary file 1 [file viruses-09-00124-s001.pdf]

Supplementary Materials:

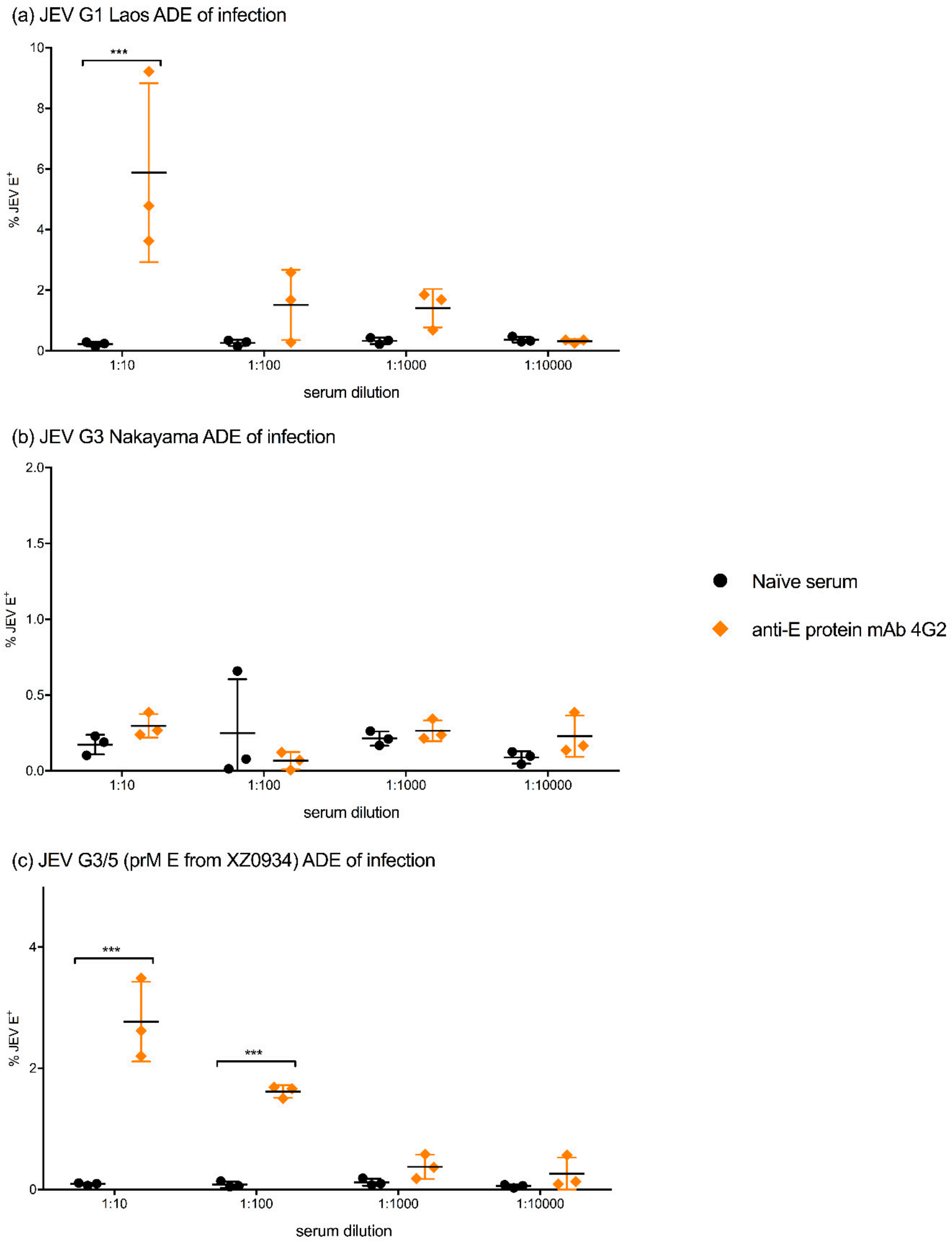

Figure S1. ADE of infection in murine macrophages J744A.1 cells. Anti-flavivirus E protein mAb 4G2 was tested for ADE activity in the murine macrophages J744A.1 cell line. ADE of infection was tested as described in figure 1 using ten-fold dilutions of the mAb instead of immune serum and for (**a**) JEV G1 Laos, (**b**) JEV G3 Nakayama and (**c**) JEV G5/G3, representing a chimeric virus expressing a G5 prM/E. The percentage of infected cells was determined after 24h. Statistical significance was calculated using a two-way Anova followed by Dunnets's multiple comparison. The results are representative of triplicate cultures repeated in two independent experiments. * $p<0.05$, ** $p<0.002$, *** $p<0.001$.
